# Clinical trials targeting neurofibromatoses-associated tumors: a systematic review

**DOI:** 10.1093/noajnl/vdac005

**Published:** 2022-01-16

**Authors:** Gabriel Roman Souza, Ahmed Abdalla, Daruka Mahadevan

**Affiliations:** Institute for Drug Development, Division of Hematology and Medical Oncology, Mays Cancer Center, University of Texas Health San Antonio MD Anderson Cancer Center, San Antonio, Texas, USA

**Keywords:** clinical trials, neurofibromatosis type 1, neurofibromatosis type 2, schwannomatosis, therapies

## Abstract

**Background:**

There is a paucity of literature that comprehensively analyzes previous and current clinical trials targeting neurofibromatoses-related tumors. This article aims to provide readers with drug development efforts targeting these tumors by analyzing translational and clinical findings.

**Methods:**

This systematic review was written according to the PRISMA guidelines. Inclusion criteria were clinical trials involving patients with neurofibromatosis type 1, type 2, or schwannomatosis that were treated with therapies targeting neurofibromatoses-associated tumors and that were registered on clinicaltrials.gov. In addition, a search was performed in PubMed, Web of Science, Google Scholar, and Embase European for articles fully describing these clinical trials.

**Results:**

A total of 265 clinical trials were registered and screened for eligibility. Ninety-two were included in this systematic review involving approximately 4636 participants. The number of therapies analyzed was more than 50. Drugs under investigation mainly act on the MAPK/ERK and PI3K/AKT/mTOR pathways, tumor microenvironment, or aberrantly over-expressed cell surface receptors. Selumetinib was the most effective medication for treating a neurofibromatosis type 1-associated tumor with approximately 68%–71% partial response for inoperable or progressive plexiform neurofibromas in children 2 years of age and older and bevacizumab for a neurofibromatosis type 2-related tumor with approximately 36%–41% partial response for vestibular schwannomas in patients 12 years of age and older.

**Conclusions:**

This systematic review presents the results of previous clinical investigations and those under development for neurofibromatoses-associated tumors. Clinicians may use this information to strategize patients to appropriate clinical trials.

**Key Points**

- This systematic review analyzes 92 clinical trials in neurofibromatoses-associated tumors.
- Over 50 therapies were or are under investigation, however, only a few have been shown to be effective and well-tolerated.

**Importance of the Study**

This systematic review comprehensively analyzes clinical trials in neurofibromatoses-associated tumors registered on clinicaltrials.gov. A total of 92 clinical trials and over 50 therapies are reviewed to provide clinicians with a better understanding of the state of the art of current treatments, lessons from past studies, future therapies in development, and strategize patients to appropriate clinical trials. We also address challenges to implementing clinical trials.

There are 3 clinically and genetically distinct forms of neurofibromatoses (NFs): neurofibromatosis types 1 (NF1) and 2 (NF2) and schwannomatosis. In September 2021, a new international consensus decided that Legius syndrome, an NF1-like syndrome, would not be classified as NFs due to the absence of neurofibromas. The general nomenclature may change in the future.^1^

NFs are autosomal dominant tumor-prone syndromes. The birth incidence is approximately 1 in 2600–3000 individuals for NF1,^2,3^ 1 in 25 000 for NF2,^4^ and 1 in 125 000 for schwannomatosis.^5^

The most common manifestations of NF1 are café-au-lait macules, cutaneous neurofibromas (CNs), plexiform neurofibromas (PNs), optic pathway gliomas (OPGs), lentiginous macules, iris Lisch nodules, and skeletal abnormalities.^1^ There is also a propensity to develop a variety of other malignancies, including astrocytic tumors, malignant peripheral nerve sheath tumors (MPNSTs), juvenile myelomonocytic leukemias, gastrointestinal stromal tumors (GISTs), breast cancers, pheochromocytomas, duodenal carcinoids, and rhabdomyosarcomas.^6^

Neurofibromatosis type 1 is due to a mutation in the tumor-suppressor gene *NF1* located on the long (q) arm of chromosome 17, at band 11.2 (17q11.2), resulting in loss of production or reduced function of neurofibromin, a RAS GTPase-activating protein (RAS-GAP) that normally negatively regulates Ras activation. GTP-bound Ras activates multiple signalizing pathways, including mitogen-activated protein kinase/extracellular signal-regulated kinase (MAPK/ERK) pathways, mammalian (mechanistic) target of rapamycin (mTOR), and stem cell factor (SCF)/c-KIT signaling.^7^

Neurofibromatosis type 2 is due to a mutation in the tumor-suppressor gene *NF2* located on the long (q) arm of chromosome 22, at band 12.2 (22q12.2). *NF2* gene generates merlin, also known as schwannomin, a cytoskeletal protein that functions as a tumor suppressor. Clinical features include bilateral vestibular schwannomas, meningiomas, and spinal tumors. Loss of merlin activates signaling pathways, including Ras/Raf/MAPK/ERK, phosphatidylinositol-3-kinase (PI3K)/AKT/mTOR, nuclear factor kappa-light-chain-enhancer of activated B cells (NFκB), and Hippo signaling^8^ (Figure 1).

**Figure 1.**
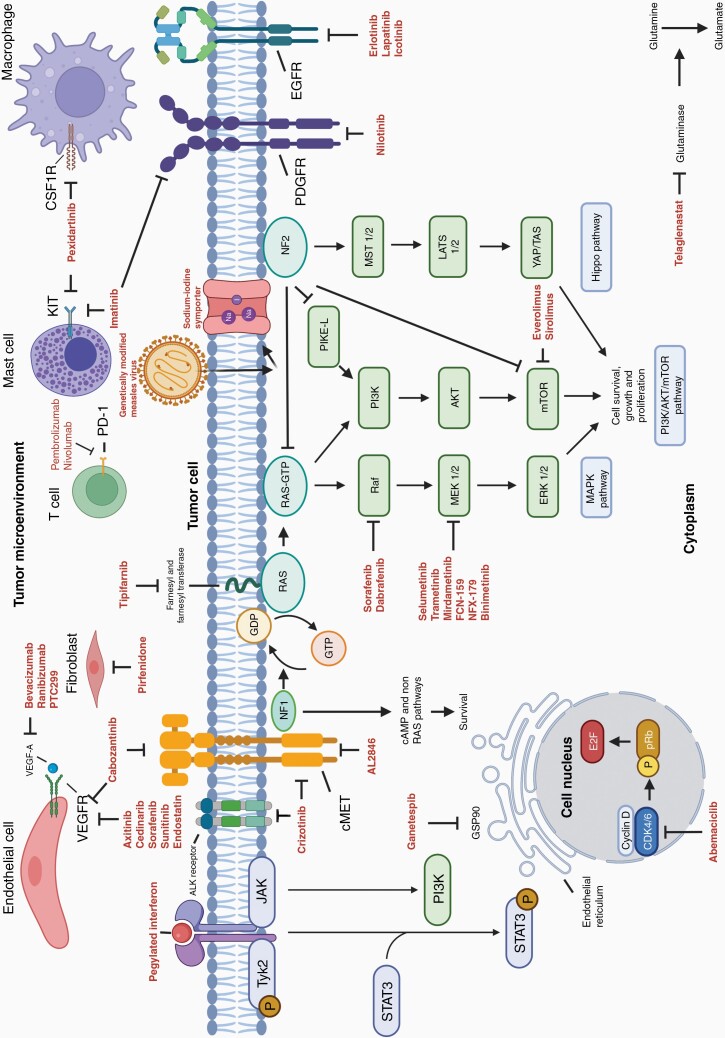
Main regulatory pathways involved in *NF1* and *NF2* mutations associated tumorigenesis with therapies under evaluation. Mutations in the *NF* suppressor genes cause loss of negative regulation in multiple pathways, leading to cancer cell proliferation, growth, and survival. Current drugs are not specific in targeting pathologic mechanisms. MEK and BRAF inhibitors act on the MAPK pathway, mTOR inhibitors on the PI3K/AKT/mTOR pathway, and farnesyl transferase inhibitors disrupt RAS signaling. Inflammation contributes to tumor development and progression and is potentiated with the recruitment of various cells, including endothelial cells, mast cells, macrophages, and fibroblasts. VEGFR, VEFG-A, KIT, and CSF1R inhibitors block the microenvironment inflammatory cells activity. There is also the possibility of evoking an immune response with interferon and potentially utilizing immune checkpoint therapies (pembrolizumab, nivolumab). Other anti-tumorigenesis mechanisms include inhibiting aberrantly over-expressed cell surface receptors (MET, KIT, PDGFR, EGFR), slowing metabolism with glutaminase inhibitors, and accumulation of unfolded proteins in the endoplasmic reticulum lumen with heat shock protein 90 inhibitors. A novel approach uses the injectable measles virus Edmonston vaccine strain engineered to express the human sodium–iodide symporter and induce cell death.^71^.

Schwannomatosis is characterized by multiple non-cutaneous peripheral and intracranial schwannomas in the absence of vestibular schwannomas. Mutations in the *SMARCB1* (22q11.23) or *LZTR1* (22q11.21) tumor-suppressor genes are present in most patients; however, they are insufficient to cause schwannomatosis and require additional somatic mutations. In *SMARCB1 (INI1)* mutation-positive schwannomas, additional genetic alterations can occur, including losing one copy of chromosome 22 and inactivating mutations in the *NF2* gene. This suggests a 4-hit, 3-step model of tumorigenesis, in which the mutated germline *SMARCB1* gene copy is kept in the tumor (hit 1), the totality or part of chromosome 22 containing the wildtype *SMARCB1* gene copy and a wildtype copy of the *NF2* gene is lost (hits 2 and 3), followed by an alteration in the remaining wildtype *NF2* gene copy (hit 4). The same model can be applied for *LZTR1* mutation-positive schwannomas. *SMARCB1* and *LZTR1* interact with histone deacetylase 4, which may represent a pathway for future targeted therapies^9^ (Figure 2).

**Figure 2.**
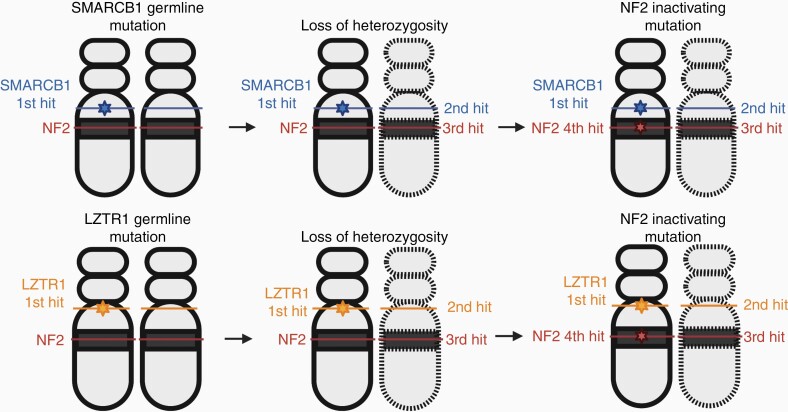
Four hit/3-step model of tumorigenesis in schwannomatosis. The mutated germline *SMARCB1* or *LZTR1* gene copy is kept in the tumor (hit 1), the totality or part of chromosome 22 containing the wildtype *SMARCB1* or *LZTR1* gene copy and a wildtype copy of the *NF2* gene is lost (hits 2 and 3), followed by a somatic mutation in the remaining wildtype *NF2* gene copy (hit 4).^9^.

In April 2020, selumetinib, a MEK inhibitor, was the first drug approved by the US Food and Drug Administration (FDA) for the treatment of an NF1-associated tumor. Currently, there are no drugs approved for NF2-associated tumors or schwannomatosis.

Clinical trials have focused on drugs that may block directly or indirectly pathways that are overactivated by *NF1* or *NF2* tumor-suppressor mutations. This systematic review will provide a comprehensive analysis of past and current clinical investigations and potential new therapeutics for NF-associated tumors.

## Materials and Methods

This systematic review was written according to the Preferred Reporting Items for Systematic Reviews and Meta-Analysis (PRISMA) 2020 guidelines. Inclusion criteria were clinical trials involving patients with neurofibromatosis type 1, type 2, or schwannomatosis that were treated with therapies targeting NF-associated tumors and that were registered on clinicaltrials.gov with the keyword neurofibromatosis. Clinical trials were ultimately selected by analyzing the detailed description. If there was uncertainty about the recruitment of individuals with NF due to ample inclusion criteria, the trial was excluded. In addition, a search was performed in PubMed, Web of Science, Google Scholar, and Embase European on November 18, 2021, for original articles in the English language describing these clinical trials in depth. Two authors (G.R.S. and A.A.) independently screened all articles and clinical trial registers. Articles were selected by title and abstract. In case of doubt, a search was made in the manuscript to find the clinical trial identifier or further details that indicated the specific study. Clinical trials were selected by title. If uncertain, a search was made in the study record detail section. Discrepancies were advanced to a third Author (D.M.) to decide for inclusion. Data were collected using a spreadsheet with predefined fields: drug, trial design, age, registered number of participants, completion date, sponsor, status, results, and clinical trial identifier. To keep this review updated, we performed a new search on clinicaltrials.gov on December 12, 2021, and 2 new trials were added.

## Results and Discussion

**Figure 3.**
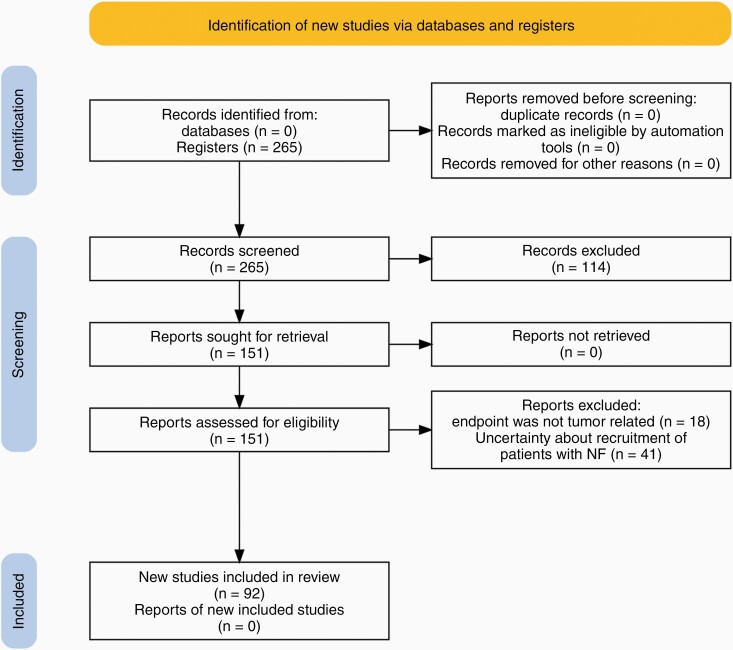
PRISMA flow diagram.

A total of 265 clinical trials from clinicaltrials.gov were registered and screened for eligibility. Ninety-two were included (Figure 3) in this systematic review involving approximately 4636 participants. We counted 2765 participants in 33 active clinical trials in NF1-associated tumors (Supplementary Table 1), while 888 participants were involved in 36 previous trials (Supplementary Table 2). We calculated that 655 patients are in 8 active clinical trials in NF2-related tumors, while 180 participated in 13 previous studies. The only clinical trial involving patients exclusively with schwannomatosis aims to recruit 46 participants. A clinical trial with antigen-specific T cells aims to recruit 100 participants with NF1, NF2, or schwannomatosis; however, the proportion of patients with each condition is unknown (Supplementary Table 3). The number of therapies analyzed in this review was more than 50. Drugs under investigation mainly act on the MAPK/ERK and PI3K/AKT/mTOR pathways, tumor microenvironment, or aberrantly over-expressed cell surface receptors (MET, KIT, PDGFR, and EGFR). Novel approaches such as using the injectable measles virus Edmonston vaccine strain engineered to express the human sodium–iodide symporter and induce cell death are under investigation.

Selumetinib was the most effective medication for treating an NF1-associated tumor with approximately 68%–71% partial response (PR) for inoperable or progressive PNs in children 2 years of age and older^10,11^ and bevacizumab for an NF2-related tumor with about 36%–41% PR for vestibular schwannomas on patients 12 years of age and older.^12,13^

This study has limitations. We had to limit clinical trials to those registered in clinicaltrials.gov with the keyword neurofibromatosis given the magnitude of information. Various clinical trials considered patients with NF eligible; however, their inclusion criteria were ample and recruitment was uncertain, so we excluded them. Not all articles linked their publication to the clinical trial identifier or mentioned it, making it challenging to understand which trial was addressed. When investigating outcomes, many trials did not publish their results or did it partially or unsatisfactorily. Therefore, it was not possible to describe their methods and results precisely. Some clinical trials only had partial results published as abstracts in scientific meetings. Finally, the definition of PR varied between trials and a comparison of results was not straightforward.

It is also important to highlight that the rarity of NF syndromes and the variety of tumors has posed challenges for the design of effective clinical trials. They require large, multicenter studies for patient accessibility. Tumors also need appropriate validated methods and endpoints to assess not only survival and radiographic progression, but also clinical outcome assessments, which require trained personnel. These are composed of the patient-reported outcome that captures symptoms that cannot be directly measured such as pain and quality of life, the clinician-reported outcome that uses the physician’s assessment to determine the fitness of the patient to be on therapy, observer-reported outcome incorporating the caregiver’s insight, and performance outcome mainly applied for neurocognitive functions.^14^

In the next session, a comprehensive description of clinical trials will provide clinicians and scientists with the current state of the art of therapies in NFs-associated tumors.

### Description of Clinical Trials Targeting Neurofibromatosis Type 1-Associated Tumors

#### Inoperable or progressive PNs.

—PNs are benign peripheral nerve tumors that occur in approximately 50% of individuals with NF1 and constitute a significant cause of morbidity and disfigurement. They also increase mortality by complications such as compression of the airway or spinal cord and transformation into MPNST.^15^

A retrospective analysis of 520 children with NF1 showed that patients with known/symptomatic PN had higher mortality (5/154, 3.2%) compared with those without (2/366, 0.5%; *P* = .024). The causes of death were MPNST (3/5), hypovolemic shock caused by a massive hemothorax from a large thoracic PN (1/5), and respiratory failure attributable to airway compression (1/5). There was a significantly higher prevalence of macrocephaly (63% versus 30%), headaches (61.6% versus 39.8%), scoliosis (41.5% versus 19.9%), optic gliomas (24% versus 15%), neuroblastoma (2% versus 0.5%), and MPNSTs (1.9% versus 0%) in the PN group. Patients underwent surgery most commonly due to neurologic deficits (32%, 25/78), physical disfigurement (31%, 24/78), orthopedic complaints (26%, 20/78), and airway difficulties (11%, 9/78). Forty-three percent of these patients needed a second resection and 18% had permanent sequelae. Given that PNs have a high risk of complications and surgical resection frequently results in added morbidity, there is a need to develop medical therapies that can halt tumor growth or cause shrinkage for a better surgical resection.^15^

PNs have more clinical trials than any other NF-related tumors. To facilitate understanding the variety of drugs under investigation, the next section is divided by therapeutic targets.

#### Targeting neoplastic cells through the mitogen-activated protein kinase pathway.

In April 2020, selumetinib, an oral selective mitogen-activated protein kinase 1 and 2 inhibitor, was the first drug approved by the FDA for neurofibromatosis type 1. The drug is currently used to treat pediatric patients 2 years of age and older with NF1 who have symptomatic, inoperable PNs.

In a phase I clinical trial of 24 children with inoperable PN, selumetinib 20–30 mg/m^2^ orally twice daily in 28-day cycles on a continuous dosing schedule showed a decreased PN volume in all participants with a median response of 31%. A PR, defined as a 20% or greater decrease in tumor volume from baseline for at least 4 weeks, was confirmed in 71% (17 of 24) of patients and was maintained for the duration of the study (median of 30 cycles) in 88% (15 of 17) of these patients. The maximum tolerated dose was 25 mg/m^2^, but a similar response was observed in the 20 mg/m^2^ group. There was slow tumor regrowth after maximal response, which could indicate treatment resistance or the result of dose reductions with a dose-dependent effect of selumetinib on PN.^10^

In the phase II trial, 50 children received selumetinib 25 mg/m^2^ orally every 12 hours in 28-day cycles on a continuous dosing schedule. PR observed was 68% (34 of 50 children), consistent with the results of the phase I trial. Fifty-six percent (28) of patients had a PR lasting at least 12 months, and the median change in neurofibroma volume at best was −27.9%. Progression-free survival (PFS) was 84% as of 3 years after starting treatment versus 15% in the National Cancer Institute natural history study (NCT00924196). Sixty-eight percent had improvement in at least one of the various functional, patient-reported, and observer-reported outcome measures. There was a significant decrease in child-reported tumor pain intensity after 2 months of treatment and child-reported and parent-reported pain interference after 4 months. The pain interference index, which measures the interference of pain in daily functioning, decreased significantly for 38% of children and 50% of parents. Quality of life measured by the Pediatric Quality of Life Inventory Generic Core Scales showed reported clinically meaningful increases in 48% of children and 58% of parents after 1 year of treatment. Fifty-six (10 of 18) percent of children with motor dysfunction experienced a clinically significant increase in strength and 38% (10 of 26) in the range of motion. Forty-four (7 of 16) percent had clinically meaningful improvement in airway impairment. Ten percent (5 of 50) of patients had a grade 3 or 4 adverse event that led to treatment discontinuation and 28% (14 of 50) required dose reductions due to toxicity.^11^ Based on these findings, selumetinib received FDA approval.^16^

Selumetinib is currently being studied for PN treatment in adults in a phase III randomized, double-blind, placebo-controlled trial that aims to recruit 146 participants. Given the efficacy of the drug in children and the power of this study, it is expected that it might expand the approval of selumetinib to adults. In addition, a phase I/II study with 30 children is investigating intermittent dosing (given twice daily on 5 out of every 7 days) of selumetinib to mitigate toxicity but maintain efficacy, while a phase I trial recruiting 20 participants aims to confirm the appropriate dose of selumetinib with a low-fat diet to support labeling statements.

The effects of selumetinib in spinal neurofibromas were studied in a retrospective analysis of individuals enrolled in the pediatric (NCT01362803) and adult (NCT02407405) phase II trials. Twenty-four patients who underwent serial spinal MRIs were included and 23 completed 12 cycles of treatment. Seventy-five percent (18 of 24) showed improvement of spinal neurofibroma burden, 21% (5 of 24) remained stable, and none worsened.^17^

The MEK inhibitor mirdametinib showed to be well-tolerated in a phase II trial. Forty-two percent of participants (8 of 19) with PN experienced a 20% or more decrease in tumor volume. There was a significant decrease in pain intensity, pain interference, and total functioning in the PR group. However, there was no change in the quality of life, one patient developed 2 treatment-related grade 3 adverse events and 5 required dose reductions.^18^ Another phase II trial is in progress with planned 100 participants.

FCN-159, a highly active MEK 1/2 inhibitor, synthesized based on the structure of trametinib, is undergoing a phase I dose-escalation and phase II dose-expansion trial with planned 160 participants. Other MEK inhibitors under investigation include binimetinib and trametinib^19^ in phase II studies involving 40 and 15 participants respectively.

#### Targeting neoplastic cells through the mammalian target of rapamycin pathway.

Everolimus, a selective mTOR inhibitor, was investigated in 2 clinical trials with mixed results. The first trial enrolled 22 participants, 17 completed the trial, and 5 withdrew due to adverse events. A significant reduction in lesion surface volume, defined as 2 standard errors less than baseline volume, was observed in 13% (4 of 31) of lesions from 19% (3 of 16) of patients.^20^ In the second trial with 23 participants, 17% (4) discontinued due to adverse events, 9% (2) for disease progression, and 9% (2) for personal convenience. The primary endpoint was a 30% reduction in target lesion volume without an increase in the target volume of 4 other lesions after 1 year of treatment. Unfortunately, none of the participants achieved the endpoint.^21^

Sirolimus was investigated in a phase II clinical trial with 46 evaluable participants for response. Twenty-four percent (11) of subjects experienced a grade 3 adverse event and 2% (1) a grade 4, respectively. The estimated median time to progression (TTP) of subjects receiving sirolimus was 15.4 months, significantly longer than 11.9 months compared to the placebo arm. No subjects achieved a PR.^22,23^

#### Targeting neoplastic cells and microenvironments with tyrosine kinase inhibitors.

Imatinib mesylate (IM) showed PR on an intent-to-treat basis in 17% of patients (6 of 36) and 26% of evaluable individuals (6 of 23). Thirty percent (7 of 23) reported subjective improvement in disease symptoms. Two patients experienced grade 3 neutropenia and one grade 4 transaminitis.^24^ A clinical trial of imatinib on patients with airway tumors was registered, however, was withdrawn given difficulties in the recruitment phase.

The multitarget receptor tyrosine kinase inhibitor cabozantinib is being investigated in a phase II, open-label, non-randomized Simon 2-stage clinical trial. The study has 2 cohorts. The pediatric cohort with patients from 3 to 15 years of age is currently recruiting, and the adult cohort study with patients 16 years of age and older was completed. The adult cohort showed PR in 42% of evaluable participants (8 of 19) who also experienced a significant reduction in pain intensity and pain interference in daily life. However, there was no change in global quality of life scores, 11 grade 3 adverse events occurred in 8 patients, and 9 required dose reductions or discontinuation. This agent has activity against c-KIT, VEGFR2, RET, FLT3, MET, and the TAM family receptors (AXL, MERTK, and TYRO3). In PN, c-KIT has a role in tumor initiation and VEGF in tumor neoangiogenesis within the microenvironment. The other targets have known activity in other tumors.^25^

A phase I trial with sorafenib, a CRAF, BRAF, and receptor tyrosine kinases inhibitor of VEGFR-2,3, PDGFR-β, c-kit, and Flt3, showed disappointing results. Nine children from 6 to 12 years of age with NF1 and inoperable PN were enrolled. The initial dose was 115 mg/m^2^. Two patients withdrew during the first cycle for non-dose-limiting toxicities, one for grade 2 hypertension, and one for grade 2 facial PN pain. Of the remaining patients, one developed grade 3 pain in his abdominal, pelvic, and thigh PN, another one grade 3 facial tumor pain which resolved after sorafenib was held, however, returned upon resuming it. The drug was then de-escalated to the 80 mg/m^2^ dose level, which is around 40% of the maximum tolerated dose for pediatric solid tumors. One patient developed grade 3 rash and another one with bipolar disorder had grade 4 mood alteration. There was no tumor shrinkage observed. Of the 6 assessable patients, 2 had PD and 1 clinical progression. Three of 4 evaluable patients had deterioration of baseline quality of life.^26^

Pexidartinib, a CSF-1R selective tyrosine kinase inhibitor, is in a phase II study and aims to recruit 81 children with refractory leukemia and solid tumors, including NF1 PN. The phase I trial of 12 participants showed that pexidartinib was tolerated at all dose levels.^27^

#### Targeting microenvironments.

In a phase I clinical trial, pegylated interferon showed PR in 29% of participants (5 of 16).^28^ The phase II clinical trial showed PR in 5% of patients (4 of 82 evaluable) and improvement in TTP, 29.4 months versus 11.8 for placebo.^29^

Tipifarnib, a farnesyltransferase inhibitor, showed to be well-tolerated in a phase I trial^30^; however, there was no significant difference in TTP or objective response compared to placebo in a phase II trial with 62 participants.^31^

Pirfenidone, a fibroblast inhibitor, was well-tolerated in a phase I trial in which dose-limiting toxicities were observed in 17% (2 of 12 patients) with 500 mg/m^2^/dose which was considered pharmacokinetically comparable to the active adult dose.^32^ In the phase II trial with 24 participants, 4 had a PR (17%), 3 had progression (12%), and 17 (71%) stable disease (SD).^33^ A subsequent phase II trial with 36 patients showed no objective response.^34^

#### Malignant peripheral nerve sheath tumors.

—MPNSTs, previously known as neurofibrosarcomas, are lethal tumors that originate in nerve sheath cells, usually within preexisting plexiform or nodular neurofibromas. The annual incidence in patients with NF1 is approximately 1.6 per 1000, and the lifetime risk is 8%–13%.^35^ A meta-analysis of more than 1800 patients with MPNST showed a significantly higher odds ratio for overall survival and disease-specific survival in the non-NF1 group than in the NF1 group; however, in the last decade, this difference has decreased. Five-year overall survival for all patients was 44%, disease-specific survival 46%, and disease-free survival 37%. Although adjunctive chemotherapy and radiation therapy can be used in selected situations when wide excision margins by surgery alone are not feasible, they do not impact mortality.^36^ Currently, no medications are approved for this type of tumor that manifests with a high growth rate and metastatic potential.

The combination of sirolimus plus ganetespib (heat shock protein 90 inhibitor) was well-tolerated; however, it failed to show responses for MPNSTs in a phase I/II trial with 20 patients. Most patients had metastatic disease (90%) and prior therapy including surgery, chemotherapy, and radiation therapy.^37^

Everolimus plus bevacizumab (VEGF inhibitor) was evaluated in a clinical trial with 25 participants. The combination did not reach the target endpoints and was not considered active in refractory MPNSTs. Twelve percent (3 of 25) of participants achieved SD.^38^ The combination of Sirolimus plus Selumetinib is in a phase II study aiming to recruit 21 participants.

The glutaminase inhibitor telaglenastat hydrochloride is under investigation in the BeGIN Study, a phase II clinical trial recruiting 108 participants with specific genetic mutations including NF1-associated solid tumors or MPNSTs with metastatic or unresectable disease.

The c-met selective tyrosine kinase inhibitor AL2846 is currently in a phase I/II clinical trial involving 192 adult patients. Primary endpoints are adverse events and objective response rates.

A novel phase I trial is investigating an approach utilizing the antitumor properties of the genetically designed injectable measles virus Edmonston vaccine strain (MVEdm) engineered to express the human sodium–iodide symporter (MV-NIS) on MPNST. A study in mice resulted in significant regression of tumor and improved survival.^39^

#### Cutaneous neurofibromas.

—CNs are benign peripheral nerve sheath tumors that occur in more than 99% of individuals with NF1 and do not carry a risk of malignant transformation, but often they represent a major cosmetic problem.^40^ The treatment consists of surgical resection of symptomatic tumors that are refractory to medical therapy. There are no current anti-neoplastic medications approved. Most clinical trials focus on topical drugs given the easy access to the tumor and better adverse events profile.

A phase II study with topical liquid diclofenac following laser microporation of CN showed no significant alterations regarding the presence of tissue necrosis, size, or histopathological features of neurofibromas in 6 participants.^41^

A phase I clinical trial had positive results with aminolevulinic acid (ALA) and red-light photodynamic therapy for CN. TUNEL assay (DeadEnd Colorimetric TUNEL System; Promega) evaluation showed 42.5 ± 19.9 apoptotic cells per visual field for ALA-treated and 1.1 ± 1.4 for vehicle-treated tumors (*P* = .002). There were no adverse events.^42^ Currently, a phase II trial is on course with 30 participants.

NPC-12G gel containing 0.2% sirolimus is undergoing a phase III randomized, double-blind, vehicle-controlled, parallel-group dose–response study with 100 participants. A phase I trial was completed but results were not published. Deoxycholic acid will be investigated in a phase I trial that is not recruiting yet.

NFX-179 gel, a MEK inhibitor, underwent a phase I trial, and the results are under review. A phase III open-label, uncontrolled, multicenter study with 168 patients is ongoing to evaluate the safety and efficacy of long-term treatment (52 weeks or more).

Selumetinib is the only oral drug under investigation for patients with NF1 and CNs. It is currently recruiting 24 patients for a phase II trial.

High-intensity ultrasonography is currently undergoing multiple clinical trials for the treatment of various solid tumors. A new phase I study will investigate this noninvasive therapy performed by interventional radiologists for ablation of CNs.

#### Low-grade gliomas.

—Low-grade gliomas (LGGs) are the most common central nervous system tumors in children with and without NF1 mutations. NF1-related gliomas typically affect the optical pathway causing vision loss and are more often multifocal than in individuals without the disease. Approximately 20% of patients develop optical pathway gliomas, and only one-third of those require treatment.^43^ The first-line treatment in pediatric patients consists of carboplatin plus vincristine or vinblastine monotherapy.^44^ Radiation is avoided due to the increased risk of secondary malignancy^45^ and moyamoya.^46^ In adults, the standard of care includes maximal safe surgical resection followed by ± chemotherapy or radiation according to the National Comprehensive Cancer Network guidelines.

The prognosis of LGGs is age-dependent. A study with 1202 pediatric patients with LGG showed a 20-year OS rate of 90.1%. In the NF1 cohort, a total of 4 deaths occurred, 2 unrelated to tumor or treatment and 2 due to malignant transformation of tumors in patients who received upfront radiotherapy. The 20-year OS was 95.1%.^47^ Another study with 339 adult patients showed a Kaplan–Meier median OS of 15.7 years. Adults also have a higher chance of presenting with high-grade tumors.^48^

A phase II trial investigated vinblastine in 54 pediatric patients with unresectable or progressive therapy-naïve LGG, 24% (13 of 54) had NF1. This trial (NCT00575796) did not include the keyword neurofibromatosis on clinicaltrials.gov; however, we decided to include it given its importance. Vinblastine was given once a week at a dose of 6 mg/m^2^ intravenously over a period of 70 weeks. Two percent of participants had complete response (CR) (1 of 54), 17% (9 of 54) PR, 7% (4 of 54) minor response (MR), 59% (32 of 54) SD, and 11% (6 of 54) progressive disease (PD), for a total response rate (CR + PR + MR) of 25.9% (14 of 54 patients). SD was achieved in 87% (47 of 54) of patients. After a median follow-up of 5 years, 57% (31 of 54) did not require any further treatment. Five-year overall survival was 94.4%, and PFS was 53.2%. There was an improvement of visual acuity in 20% (5 of 25) of patients with OPGs, stable vision in 56% (14 of 25), and deterioration in 16% (4 of 25). In 2 patients, the vision was normal throughout treatment and for one information was missing. Five-year PFS for patients older than 10 years of age was similar to the younger. In the NF1 group, 15% (2 of 13) percent had PR, 15% (2 of 13) MR, 69% (9 of 13) SD, 15% (2 of 13) PD, for a response rate of 30% (4 of 13). They were younger at diagnosis with a median age of 3.8 years compared to 7.1 in the non-NF1 group. Five-year PFS was significantly higher at 85.1% versus 42% in the non-NF1. Forty-one percent (22 of 54) of patients experienced grade 3 neutropenia, 35% (19 of 54) grade 4, 11% (6 of 54) grade 3 febrile neutropenia, 9% (5 of 54) grade 3 infections (zoster, varicella, *Staphylococcus aureus*, influenza H1N1, and pneumonia), and 4% (2 of 54) grade 3 anemia. Unfortunately, there was no breakdown of data for NF1 patients in this section.^44^

A phase II study of continuous oral everolimus for recurrent radiographic-progressive NF1-associated pediatric LLG showed a response in 68% of participants (15 of 22), defined as either reduction (1 CR, 2 PR) or stabilization of tumor growth (12 SD). Of these, 10 of 15 remained free of progression.^49^

A phase II clinical trial with sorafenib of 12 pediatric participants with LGGs was terminated due to rapid and unexpectedly high tumor progression rate. One participant developed transaminase elevation after one week of treatment and by protocol, the therapy was stopped. She was then admitted 2 days later with sepsis requiring ventilator support. A repeat MRI 5 weeks later showed tumor growth, so the patient was not assessed for response. Of the 11 evaluable participants, 3 had NF1. PD was defined as a greater than 15% increase in tumor volume as measured by 3-dimensional volumetric tumor analysis. After 3 cycles, 82% (9 of 11) of patients (including all 3 with NF1) had PD at a median time of 2.8 months. One patient with SD decided to discontinue treatment after 7 cycles. One had PR after 3 cycles, however, discontinued treatment after 9 cycles due to recurrent hand-foot skin syndrome.^50^ This clinical trial, similarly to the clinical trial that sorafenib caused worsening of pain in PNs, highlights that nonspecific tyrosine kinase inhibitors may have paradoxical effects in tumors and should be used with caution.

A phase II trial with selumetinib of 220 patients with recurrent, refractory, or progressive LGG assigned participants to numerous strata. Stratum 3 involved patients with NF1. Forty percent (10 of 25) of patients had PR. Sixty-eight percent (17 of 25) of individuals did not have progression with a median follow-up of 48.6 months. There were 10 grade 3 toxicities and no treatment-related deaths.^51^ A phase III trial of 220 participants with Selumetinib is currently recruiting.

Many trials are ongoing. A phase III randomized, single-arm, open-label study with an estimated enrollment of 290 patients compares selumetinib versus carboplatin plus vincristine for LGG in patients with NF1 not previously treated. The primary outcome is event-free survival and visual acuity improvement. Trametinib is being investigated in combination with hydroxychloroquine in stratum 2 of a phase I/II trial aiming to recruit 75 participants. Abemaciclib, a CDK 4/6 inhibitor, is also in phase I/II clinical trial with the intention to recruit 50 participants.

A phase II trial with a synthetic complex of carboxymethylcellulose, polyinosinic-polycytidylic acid, and poly-l-lysine double-stranded RNA (poly-ICLC), a natural killer cell inducer, enrolled 6 patients with NF1 and LGG. A PR was observed in 3 of the 5 evaluable patients. In addition, the medication was well-tolerated with low-grade toxicities. Currently, there is an expansion of the phase II study.^52^

#### Other tumors.

—Patients with neurofibromatosis type 1 also have an increased risk for malignancies outside the central nervous system, including myeloid leukemias, GISTs, pheochromocytomas, breast cancers, duodenal carcinoids, and rhabdomyosarcomas.^6^

A trial with the agent selumetinib for NF1 patients with GIST was withdrawn due to slow accrual. A phase II trial is evaluating trametinib in patients with a clinical diagnosis of neurofibromatosis type 1 or *NF1* gene mutation with juvenile myelomonocytic leukemia. It intends to enroll 24 patients.

### Description of Clinical Trials Targeting Neurofibromatosis Type 2-Associated Tumors

#### Vestibular schwannomas.

—The hallmark of neurofibromatosis type 2 is vestibular schwannomas. They are present in approximately 90%–95% of patients and are typically bilateral. The most common symptoms are tinnitus, hearing loss, and vestibular balance disorder.^53^ Complications include brainstem compression and hydrocephalus. If treatment is deemed necessary, surgery remains the standard of care. In addition, some centers use radiation therapy and bevacizumab in selected patients.^54^ In the modern era, few patients with NF2 who have access to specialized neuro-otologic treatment die due to vestibular schwannomas. Operative mortality is less than 1% and recurrence rates are minimal.^55^ Therapies in development aim to halt progression, reduce these tumors to improve hearing, or improve surgical outcomes.

#### Vascular endothelial growth factor inhibitors.

Bevacizumab, a monoclonal antibody against vascular endothelial growth factor (VEGF), induced both tumor shrinkage and hearing improvement in patients with NF2-associated vestibular schwannomas in retrospective case series^56,57^ and prospective clinical studies. The mechanism of anti-VEGF therapy-induced tumor growth inhibition and hearing improvement is poorly understood.

In a meta-analysis of 8 observational studies involving a total of 161 patients with 196 assessable NF2-associated vestibular schwannomas, radiographic response to bevacizumab was PR in 41%, SD in 47%, and PD in 7%. In the subset of patients with assessable audiometric data, hearing was improved in 20%, stable in 69%, and worsened in 6%. Toxicity was seen in 17%, including one fatal intracranial hemorrhage. Surgical intervention was required in 11% of patients.^58^

In a phase II study involving 22 participants, 32% (7 of 22) achieved PR, and 41% (9 of 22) had hearing responses. Improvement of quality of life was reported in 30% of patients and tinnitus in 60%. Three grade 3 adverse events occurred in 3 participants, hypertension (n = 2), and abdominal pain (n = 1).^12^ Another study with 14 participants showed PR in 43% (6 of 14) and hearing improvement in 36% (5 of 14), respectively. To determine the durability of response, bevacizumab was stopped after 12 months. Three of 5 patients who had hearing improvement maintained it after 6 months. Three grade 3 adverse events were attributed to the drug, hypertension (n = 2), and immune-mediated thrombocytopenic purpura (n = 1).^13^

#### mTOR inhibitors.

Several trials were started based on preclinical data in mice showing that mTORC1 inhibition delayed the growth of NF2 schwannomas.^59^

There are mixed results on the efficacy of everolimus for progressive vestibular schwannomas in patients with NF2. In a phase I study with 10 participants, none of the 9 evaluable patients experienced a clinical or magnetic resonance imaging response.^60^ In a phase II trial with 10 participants, 9 were evaluable, 44% (4) had progression, and 56% (5) had SD.^61^ There is one ongoing phase II trial recruiting participants.

#### Tyrosine kinase inhibitors.

Lapatinib, a dual human epidermal growth factor receptor 2 (HER2) and epidermal growth factor receptor 1 (EGFR1) tyrosine kinase inhibitor, showed evidence of objective volumetric response in 23% of patients (4 of 17) and hearing improvement in 31% (4 of 13) with NF2-related progressive vestibular schwannoma in a phase II trial.^62^

#### Cyclooxygenase 2 inhibitor.

In a study with 15 sporadic and 15 NF2-related vestibular schwannomas, cyclooxygenase 2 (COX-2) expression was found in 29 of 30 tumors. High levels of COX-2 were associated with high proliferation.^63^ In a retrospective study with 347 patients with vestibular schwannomas, 86 had sequential scans available for 3D-segmented volumetric analysis, and 25% were taking aspirin. In the aspirin group, 32% (8) had tumor growth versus 59% (36) in the nonuser group.^64^ Based on these findings, a phase II prospective, randomized, double-blind, longitudinal study using aspirin, a nonselective cyclooxygenase inhibitor, aims to recruit 300 participants with NF2-related or sporadic vestibular schwannomas. The primary endpoint is PFS.

#### Other therapies.

AR-42, a histone deacetylase inhibitor that acts on the PI3K/AKT pathway, was investigated in a phase I, open-label, dose-escalation study that had 30% (5 of 17) of enrolled patients with NF2. SD was the best outcome in 53% (8 of 15) with evaluable responses. The established optimal dose was 60 mg 3 times weekly for 3 weeks of a 28-day cycle. A phase II clinical trial will start soon.^65^ Brigatinib, an anaplastic lymphoma kinase (ALK) inhibitor, is undergoing a large phase II clinical trial involving 80 patients, which is currently recruiting. Crizotinib, an ALK/FAK1 inhibitor, is under investigation in small phase II single-arm trials. Endostatin was also studied; but unfortunately, no results were posted. Axitinib, a VEGFR inhibitor, had results published in the 19th International Symposium on Pediatric Neuro-oncology. Seventeen percent (2 of 12) of patients experienced PR for VS and 3 hearing responses. All patients experienced drug-related toxicities. The authors concluded that axitinib was more toxic and less effective than bevacizumab and for these reasons discouraged further investigations.^66^

#### Meningiomas.

—Meningiomas are the second most common tumor in patients with NF2, with an incidence of 45%–58%.^67^ They can arise both at intracranial sites and in the spinal cord and are a considerable cause of morbidity due to seizures, paralysis, and wasting.^55^ They usually stop growing at a specific size and do not require treatment. Surgery is the treatment of choice for rapid-growing or impairing tumors according to the NCCN guidelines. If complete surgical resection is unfeasible, radiation therapy may be used.

Lapatinib was studied in a phase II trial with 21 participants with NF2-related tumors; 8 patients with 17 progressive meningiomas met the criteria for evaluation. Two tumors increased more than 20% volumetrically when on treatment, compared to 8 tumors when off treatment.^68^

In a phase II trial with 6 participants, everolimus did not cause tumor growth inhibition in any tumors but increased TTP from 5.5 months to 12 months. However, statistical significance was not calculated.^61^

Vistusertib had partial results presented in the 2021 ASCO annual meeting. Treatment of 28 patients was associated with a progression-free survival at 6 months that exceeded the Response Assessment in Neuro-Oncology target of 35% for recurrent high-grade meningiomas.^69^

A phase II open-label, parallel assessment, 4-arm clinical trial with 124 adult patients is investigating multiple agents for meningiomas with specific mutations. GSK2256098, a FAK inhibitor, is under investigation on patients with progressive meningiomas and NF2 mutation.

A phase II/III, parallel-group, 2-staged, randomized trial recently registered will investigate AR-42 in 89 patients 12 years of age or older with NF2 or *NF2*-mutated recurrent meningiomas. Cohort A will investigate 2 dose levels and cohort B will be a treatment arm and placebo arm in a ratio of 2:1.

### Description of Clinical Trials in Schwannomatosis

Multiple schwannomas are the hallmark feature of schwannomatosis. The most common symptom is pain, with 68% of patients experiencing chronic pain.^70^ Unfortunately, due to the rarity of the disease, clinical trials are scarce.

The first clinical trial involving exclusively patients with schwannomatosis started recruiting in 2020 and aims to have 46 adult participants. The use of tanezumab, an anti-nerve growth factor, will be investigated in a phase II, randomized, placebo-controlled study, addressing pain in patients receiving background non-NSAID therapy.

Antigen-specific T cells (CAR-T) and engineered immune effector cytotoxic T cells (EIE) modified by immunoregulatory genes and immune-modified dendritic cell vaccine (DCvac) are being investigated in patients with NFs (NF1, NF2, or schwannomatosis) and aim to recruit 100 participants. It is unclear how many patients will have schwannomatosis.

## Conclusions

The recent approval of selumetinib, a MEK inhibitor, for the treatment of PNs in children with NF1 is an important landmark of an effective therapeutic target in NF. The drug is now under investigation in adults, and there was an expansion of clinical trials. Selumetinib was the most efficacious drug for treating an NF1-associated tumor with approximately 68%–71% PR for inoperable or progressive PNs in children 2 years of age and older and bevacizumab for an NF2-related tumor with about 36%–41% PR for vestibular schwannomas in patients 12 years of age and older. Schwannomatosis does not have much data available given the rarity of the disease, but the first clinical trial was initiated. Promising novel therapies include using the antitumor properties of the genetically engineered injectable measles virus Edmonston vaccine strain, Antigen-specific T cells, engineered immune effector cytotoxic T cells modified by immunoregulatory genes, and immune-modified dendritic cell vaccine (refer to Table 1 for a summary of selected clinical trials). Given the magnitude of information available, it was necessary to summarize these findings systematically so that clinicians may strategize patients to appropriate clinical trials.

**Table 1.** Selected Clinical Trials Targeting Neurofibromatoses-Associated Tumors

Drug	Mechanism of Action	Trial Design	Age	Enrollment	Status	Results	Clinical Trial Identifier
*NF1 plexiform neurofibromas*							
Cabozantinib	Multitarget tyrosine kinase inhibitor	Phase II trial, open-label, nonrandomized Simon 2-stage study	16 years and older	45	Completed for the cohort of patients older than 16 years of age. Recruiting for 3–15 years of age.	PR in 42% (8 of 19 evaluable participants) with improvement in pain intensity and pain interference in daily life^25^	NCT02101736
Mirdametinib	MEK inhibitor	Phase II trial, single-arm, open-label	16 years and older	19	Completed	PR in 42% (8 of 19 patients). Significant decrease in pain intensity, pain interference, and total functioning in the PR group^18^	NCT02096471
Selumetinib	MEK inhibitor	Phase I/II trial, nonrandomized, single-arm, open-label	2 years to 18 years	99	Active, not recruiting	Phase I: PR in 71% (17 of 24) of patients maintained for 30 months in 88% (15 of 17). Phase II: PR in 68% (34 of 50 children). PFS was 84% as of 3 years versus 15% in the natural history study^10,11^	NCT01362803
Selumetinib	MEK inhibitor	Phase III trial, randomized, double-blind, placebo-controlled, 2-arm	18 years and older	146	Recruiting	In progress	NCT04924608
*NF1 malignant peripheral nerve sheath tumors*							
Measles virus Edmonston vaccine strain (MVEdm)	Infects and destroys tumor cells	Phase I trial, single-arm, open-label	18 years and older	30	Recruiting	In progress	NCT02700230
*NF1 cutaneous neurofibromas*							
NFX-179 gel	MEK inhibitor	Phase II trial randomized, double-blind, vehicle-controlled, parallel-group	18 years and older	168	Recruiting	In progress	NCT05005845
NPC-12G gel containing 0.2% sirolimus	mTOR Inhibitor	Phase III, open-label, uncontrolled, multicenter study	3 years and older	100	Active, not recruiting	In progress	NCT04461886
*NF1 low-grade gliomas*							
Selumetinib	MEK inhibitor	Phase I/II trial, single-arm, open-label	3 years to 21 years	220	Active, not recruiting	In progress. Partial results: PR in 40% (10 of 25 patients). 68% (17 of 25) of individuals did not have progression with a median follow-up of 48.6 months^51^	NCT01089101
Selumetinib	MEK inhibitor	Phase III randomized study, parallel assessment, open-label	2 years to 21 years	220	Recruiting	In progress	NCT04166409
Sorafenib	BRAF inhibitor and multiple tyrosine kinase inhibitors	Phase II trial, single-arm, open-label	2 years and older	12	Terminated	Terminated due to rapid and unexpectedly high tumor progression rate^50^	NCT01338857
*NF2 vestibular schwannomas*							
Aspirin	Cyclooxygenase inhibitor	Phase II, prospective, randomized, double-blind, longitudinal study	12 years and older	300	Recruiting	In progress	NCT03079999
Bevacizumab	VEGF-A inhibitor	Phase II trial, single-arm, open-label	12 years and older	14	Completed	PR in 43% (6 of 14) of patients. Hearing improvement in 36% (5 of 14)^13^	NCT01207687
Bevacizumab	VEGF-A inhibitor	Phase II trial, single-arm, open-label	6 years and older	22	Completed	PR in 32% (7 of 22) of patients. Hearing response in 41% (9 of 22)^12^	NCT01767792
*NF2 meningiomas*							
AR-42	Histone deacetylase inhibitor	Phase I trial, single-arm, open-label	18 years and older	5	Active, not recruiting	In the postdoc analysis: 6 evaluable patients had 15 tumors (8 VS, and 7 meningiomas). Tumor volume increased in 6, remained stable in 8, and decreased in 1 tumor. There were 10 grade 3 toxicities and 1 grade 4^65^	NCT02282917
AR-42	Histone deacetylase inhibitor	Phase II/III, parallel-group, 2-staged, randomized	12 years and older	89	Active, not yet recruiting	In progress	NCT05130866
*Schwannomatosis*							
Tanezumab	Anti-nerve growth factor	Phase II trial, randomized, parallel assignment, open-label	18 years and older	46	Recruiting	In progress	NCT04163419
*NF1, NF2, or schwannomatosis*							
Antigen-specific T cells CAR-T/CTL and DCvac	Immunotherapy	Phase I/II trial, randomized, parallel assignment, open-label	1 year to 80 years	100	Recruiting	In progress	NCT04085159

## Supplementary Material

vdac005_suppl_Supplementary_Tables

## Notes

**Authorship statement**. Conception: G.R.S. and D.M.; design: G.R.S. and D.M.; literature review: G.R.S., A.A., and D.M.; writing—original draft: G.R.S.; writing—review and editing: G.R.S., A.A., and D.M.; creation of figures: G.R.S.; creation of tables: G.R.S. and A.A. All authors read and approved the final manuscript.
