# Supplementary material for: Clinical trials targeting neurofibromatoses-associated tumors: a systematic review
Source: Neurooncol Adv. 2022 Jan 16;4(1):vdac005. doi: 10.1093/noajnl/vdac005 (PMC8919406; doi:10.1093/noajnl/vdac005)
Supplement: vdac005_suppl_Supplementary_Tables [file vdac005_suppl_supplementary_tables.docx]

**Supplementary Table S1. Review of ongoing clinical trials in neurofibromatosis type 1-associated tumors**

| **Drug** | **Mechanism of action** | **Trial design** | **Age** | **Estimated enrollment** | **Estimated Completion Date** | **Sponsor** | **Recruitment status** | **Clinical trial identifier** |
| --- | --- | --- | --- | --- | --- | --- | --- | --- |
| **Plexiform Neurofibromas** | | | | | | | | |
| Binimetinib | MEK inhibitor | Phase II trial, single-arm, open-label | 1 year and older | 40 | December 31, 2026 | University of Alabama at Birmingham | Active, not recruiting | NCT03231306 |
| Cabozantinib | Multi-target tyrosine kinase inhibitor | Phase II trial, open-label, non-randomized Simon two-stage study | 3 years to 15 years | 45 | December 2023 | University of Alabama at Birmingham | Active (pediatric cohort recruiting) | NCT02101736 ^25^ |
| FCN-159 | MEK inhibitor | Open-label, single-arm phase I dose-escalation, and phase II dose-expansion | 2 years to 70 years | 160 | April 30, 2025 | Shanghai Fosun Pharmaceutical Development Co, Ltd. | Not yet recruiting | NCT04954001 |
| Mirdametinib | MEK inhibitor | Phase II trial, single-arm, open-label | 2 years and older | 100 | July 31, 2022 | SpringWorks Therapeutics, Inc. | Recruiting | NCT03962543 |
| Pexidartinib (PLX3397) | KIT and CSF1R tyrosine kinase inhibitor | Phase I/II trial, non-randomized, sequential assignment, open-label | 3 Years to 35 Years | 81 | December 1, 2024 | National Cancer Institute | Recruiting | NCT02390752 |
| Selumetinib | MEK inhibitor | Phase I trial, single-arm, open-label | 3 years to 99 years | 32 | October 8, 2026 | AstraZeneca | Active, not recruiting | NCT04590235 |
| Selumetinib | MEK inhibitor | Phase II trial, single-arm, open-label | 18 years and older | 60 | January 1, 2025 | National Cancer Institute | Recruiting | NCT02407405 |
| Selumetinib | MEK inhibitor | Phase I/II trial, non-randomized, single-arm, open-label | 2 years to 18 years | 99 | January 1, 2030 | National Cancer Institute | Active, not recruiting | NCT01362803 ^11^ |
| Selumetinib | MEK inhibitor | Phase III trial,  randomized, double-blind, placebo-controlled, two-arm | 18 years and older | 146 | May 22, 2025 | AstraZeneca | Recruiting | NCT04924608 |
| Selumetinib | MEK inhibitor | Phase I trial, single-arm, open-label | 3 Years to 18 Years | 12 | September 9, 2022 | AstraZeneca | Active, not recruiting | NCT04495127 |
| Selumetinib | MEK inhibitor | Phase I trial, single-arm, open-label | 12 Years to 17 Years | 20 | February 28, 2023 | AstraZeneca | Active, not recruiting | NCT05101148 |
| Trametinib | MEK inhibitor | Phase II trial, single-arm, open-label | 1 year to 17 years | 15 | July 2022 | Region Skane | Recruiting | NCT03741101 |
| **Inoperable Plexiform Neurofibromas or Low-grade Gliomas** | | | | | | | | |
| Selumetinib | MEK inhibitor | Phase I/II trial, single-arm, open-label, intermittent schedule of drug dosing | 3 years to 18 years | 30 | December 2023 | Great Ormond Street Hospital for Children NHS Foundation Trust | Recruiting | NCT03326388 |
| Trametinib | MEK inhibitor | Phase II trial, multicentric open-label basket trial including four groups. | 1 month to 25 years | 150 (groups 1 and 2 recruiting NF1 patients) | June 1, 2026 | St. Justine’s Hospital | Recruiting | NCT03363217 ^19^ |
| Malignant Peripheral Nerve Sheath Tumors | | | | | | | | |
| AL2846 | Tyrosine kinase inhibitor selective to c-met | Phase I/II trial, single-arm, open-label | 18 years to 75 years | 192 | December 30, 2024 | Chia Tai Tianqing Pharmaceutical Group Co., Ltd. | Recruiting | NCT05011019 |
| Measles virus Edmonston vaccine strain (MVEdm) | Infects and destroys tumor cells | Phase I trial, single-arm, open-label | 18 years and older | 30 | June 15, 2022 | Mayo Clinic | Recruiting | NCT02700230 |
| Pexidartinib (PLX3397) Plus Sirolimus | KIT and CSF1R tyrosine kinase inhibitor + mTOR Inhibitor | Phase I/II trial, non-randomized, parallel assignment, open-label | 18 years and older | 43 | March 2024 | Gulam Manji | Recruiting | NCT02584647 |
| Selumetinib Plus Sirolimus | MEK inhibitor + mTOR Inhibitor | Phase II trial, open-label, non-randomized Simon two-stage study | 12 years and older | 21 | September 2021* | Sarcoma Alliance for Research through Collaboration | Recruiting | NCT03433183 |
| Telaglenastat Hydrochloride | Glutaminase inhibitor | Phase II trial, single-arm, open-label | 18 years and older | 108 | August 31, 2022 | National Cancer Institute | Recruiting | NCT03872427 |
| TQ-B3234 | MEK inhibitor | Open-label, Single-arm Phase I Dose-escalation, and Phase II Dose-expansion | 18 Years to 75 Years | 120 | December 2024 | Chia Tai Tianqing Pharmaceutical Group Co., Ltd. | Not yet recruiting | NCT05107037 |
| Cutaneous Neurofibromas | | | | | | | | |
| Aminolevulinic acid | Metabolic conversion of ALA to protoporphyrin IX | Phase II trial, single-arm, open-label | 14 years to 30 years | 30 | August 2023 | Medical College of Wisconsin | Recruiting | NCT02728388 |
| Deoxycholic acid | Disrupts cell membranes in adipocytes | Phase I trial, single-arm, open-label | 18 years to 85 years | 20 | December 2023 | Massachusetts General Hospital | Not yet recruiting | NCT04730583 |
| High-intensity focused ultrasound (HIFU) | Tumor ablation | Phase I trial, single-arm, open-label | 18 years and older | 20 | January 1, 2024 | Joergen Serup | Not yet recruiting | NCT05119582 |
| NFX-179 gel | MEK inhibitor | Phase II trial randomized, double-blind, vehicle-controlled, parallel-group | 18 years and older | 168 | February 2023 | NFlection Therapeutics, Inc. | Recruiting | NCT05005845 |
| NPC-12G gel containing 0.2% Sirolimus | mTOR Inhibitor | Phase III, open-label, uncontrolled, multicenter study | 3 years and older | 100 | April 30, 2022 | Nobelpharma | Active, not recruiting | NCT04461886 |
| Selumetinib | MEK inhibitor | Phase II trial, single-arm, open-label | 18 years and older | 24 | December 31, 2021 | National Cancer Institute | Recruiting | NCT02839720 |
| **Low-grade Gliomas** | | | | | | | | |
| Poly-ICLC | Immune stimulant, toll-like receptor-3 agonist | Phase II trial, single-arm, open-label | Up to 22 years | 20 | February 15, 2026 | University of Alabama at Birmingham | Not yet recruiting | NCT04544007 |
| Selumetinib | MEK inhibitor | Phase III Randomized Study, parallel assessment, open-label | 2 years to 21 years | 290 | May 1, 2027 | National Cancer Institute | Recruiting | NCT03871257 |
| Selumetinib | MEK inhibitor | Phase I/II trial, single-arm, open-label | 3 years to 21 years | 220 | December 1, 2025** | National Cancer Institute | Active, not recruiting | NCT01089101 (partial results available)^51^ |
| Selumetinib | MEK inhibitor | Phase III Randomized Study, parallel assessment, open-label | 2 Years to 21 Years | 220 | December 31, 2026 | National Cancer Institute | Recruiting | NCT04166409 |
| Trametinib Plus Hydroxychloroquine | MEK inhibitor + upregulation of MMP1 protein | Phase I/II trial, non-randomized, parallel assignment, open-label | 1 year to 30 years | 75 | February 28, 2027 | Pediatric Brain Tumor Consortium | Recruiting | NCT04201457 |
| **Atypical Neurofibromas** | | | | | | | | |
| Abemaciclib | CDK 4/6 inhibitor | Phase I/II trial, single-arm, open-label | 12 or older | 50 | December 1, 2023 | National Cancer Institute | Recruiting | NCT04750928 |
| **Relapsed or Refractory Juvenile Myelomonocytic Leukemia** | | | | | | | | |
| Trametinib | MEK inhibitor | Phase II trial, single-arm, open-label | 1 month to 21 years | 24 | March 31, 2024 | National Cancer Institute | Recruiting | NCT03190915 |

**Supplementary Table S2. Review of previous clinical trials in neurofibromatosis type 1-associated tumors**

| **Drug** | **Mechanism of action** | **Trial design** | **Age** | **Enrollment** | **Completion Date** | **Sponsor** | **Status** | **Results** | **Clinical trial identifier** |
| --- | --- | --- | --- | --- | --- | --- | --- | --- | --- |
| **Inoperable or Progressive Plexiform Neurofibromas** | | | | | | |  |  |  |
| Cabozantinib | Multi-target tyrosine kinase inhibitor | Phase II trial, open-label, non-randomized Simon two-stage study | 16 years and older | 45 | 2020 | University of Alabama at Birmingham | Completed for the cohort of patients above 16 years of age. Recruiting for 3 to 15 years | PR in 42% (8 of 19 evaluable participants) with improvement in pain intensity and pain interference in daily life. A total of 11 grade 3 adverse events affected 8 patients^25^ | NCT02101736 (same study is in table 1) |
| Everolimus | mTOR Inhibitor | Phase II trial, single-arm, open-label | 6 years and older | 9 | April 2015 | Novartis Pharmaceuticals | Terminated | The study was terminated due to poor patient’s accrual | NCT01365468 |
| Everolimus | mTOR Inhibitor | Phase II trial, single-arm, open-label | 18 years and older | 24 | March 2016 | The University of Texas Health Science Center, Houston | Completed | PR in 13% (4 of 31) of lesions from 19% (3 of 16) of patients^20^ | NCT02332902 |
| Everolimus | mTOR Inhibitor | Phase II trial, single-arm, open-label | 18 years to 60 years | 30 | October 2013 | Assistance Publique - Hôpitaux de Paris | Completed | None of the 23 everolimus-treated patients in the intention-to-treat population reached the primary end point^21^ | NCT01412892 |
| Imatinib mesylate | Tyrosine kinase Inhibitor | Phase I/II trial, single-arm, open-label | 3 years to 65 years | 21 | December 2016 | Indiana University School of Medicine | Completed | No indexed paper found | NCT01140360 |
| Imatinib mesylate | Tyrosine kinase Inhibitor | Phase II trial, single-arm, open-label | 3 years to 65 years | 36 | August 2012 | Indiana University | Completed | PR in 6 of 36 patients (17%, 95% CI 6-33)^24^ | NCT01673009 |
| Imatinib mesylate | Tyrosine kinase Inhibitor | Phase II trial, single-arm, open-label | 2 years to 21 years | 5 | March 1, 2019 | St. Justine’s Hospital | Terminated | Slow accrual 5 patients out of 25 expected. Primary objectives could not be met without recruiting more patients. | NCT02177825 |
| Imatinib mesylate | Tyrosine kinase Inhibitor | Phase II trial, single-arm, open-label | 6 Months to 12 Years | 0 | September 1, 2021 | Indiana University | Withdrawn | Enrollment not feasible | NCT03688568 |
| Mirdametinib (PD-0325901) | MEK inhibitor | Phase II trial, single-arm, open-label | 16 years and older | 19 | August 1, 2018 | University of Alabama at Birmingham | Completed | PR in 42% (8 of 19 patients). Significant decrease in pain intensity, pain interference, and total functioning in the PR group ^18^ | NCT02096471 |
| Nilotinib (AMN107) | Tyrosine kinase Inhibitor | Phase I trial, single-arm, open-label | 18 years and older | 6 | October 2016 | Indiana University | Completed | No indexed paper found | NCT01275586 |
| PEGylated interferon | Activation of the JAK/STAT pathway | Phase I trial, single-arm, open-label | 1 year to 21 years | 36 | January 2011 | National Institutes of Health Clinical Center | Completed | 29% (5 of 17) of patients who underwent volumetric analysis had a 15%-22% decrease in volume ^28^ | NCT00253474 |
| PEGylated interferon | Activation of the JAK/STAT pathway | Phase II trial, single-arm, open-label | 18 months to 21 years | 86 | April 2014 | University of Pittsburgh | Completed | Cumulative toxicity was not observed in the 17 NF1 patients who received a median of 10 cycles^29^ | NCT00396019 |
| Pirfenidone | Fibroblast inhibitor | Phase II trial, single-arm, open-label | 18 years to 70 years | 24 | August 2004 | Mayo Clinic | Completed | 17% (4 of 24) of patients had a decrease in tumor volume by 15% or more, 12% (3) had tumor progression, and 71% (17) remained stable^33^ | NCT00754780 |
| Pirfenidone | Fibroblast inhibitor | Phase II trial, single-arm, open-label | 3 years to 21 years | 36 | April 1, 2010 | National Cancer Institute | Completed | None of the 36 patients showed objective response^34^ | NCT00076102 |
| Pirfenidone | Fibroblast inhibitor | Phase I trial, single-arm, open-label | 3 years to 21 years | 16 | Not provided | National Cancer Institute | Completed | Dose-limiting toxicities were observed in 17% (2 of 12) of patients with 500 mg/m^2^/dose which was considered pharmacokinetically comparable to the active adult dose^32^ | NCT00053937 |
| Sirolimus | mTOR Inhibitor | Phase II trial, single-arm, open-label | 3 years to 75 years | 58 | December 2015 | University of Alabama at Birmingham | Completed | No PR. TTP 3.5 months longer with sirolimus versus placebo in 46 subjects^22,23^ | NCT00634270 |
| Sorafenib | BRAF inhibitor and multiple tyrosine kinase inhibitor | Phase I trial, single-arm, open-label | 3 years to 18 years | 9 | June 16, 2011 | National Cancer Institute | Completed | No posted results on clinicaltrials.gov | NCT00727233 |
| Sunitinib | Tyrosine kinase Inhibitor | Phase II trial, single-arm, open-label | 3 years to 65 years | 19 | February 14, 2018 | Indiana University | Terminated by FDA | 1 patient died of uncertain cause but possibly related to the drug and the study was terminated | NCT01402817 |
| Talaporfin sodium (LS11) | Direct cytotoxicity due to reactive oxygen species | Phase I trial, single-arm, open-label | 3 Years to 21 Years | 7 | July 2012 | Children's Hospital of Philadelphia | Terminated | The study was terminated due to the expiration of study materials | NCT00716469 |
| Tipifarnib (R115777) | Farnesyltransferase inhibitor | Phase II trial, randomized, flexible crossover, double-blinded, placebo-controlled trial | 3 years to 25 years | 62 | February 19, 2009 | National Cancer Institute | Completed | No objective response in 62 participants^31^ | NCT00021541 |
| Vinblastine Plus Methotrexate | Microtubule formation inhibitor + folic acid antagonist | Phase II trial, single-arm, open-label | up to 25 years | 23 | March 2016 | Children’s Hospital of Philadelphia | Completed | No indexed paper found | NCT00030264 |
| **Plexiform Neurofibroma and/or Neurofibroma Near the Spine** | | | | | | | | |  |
| Cediranib Maleate (AZD2171) | VEGFR inhibitor | Phase II trial, single-arm, open-label | 18 years and older | 26 | May 31, 2016 | National Cancer Institute | Terminated | Terminated (closed due to slow accrual before the interim analysis) | NCT00326872 |
| **Malignant Peripheral Nerve Sheath Tumors** | | | | | | |  |  |  |
| Doxorubicin hydrochloride and ifosfamide (IA) followed by etoposide and ifosfamide (IE) | Generates free radicals, intercalates in DNA + cross-links DNA + inhibits topoisomerase II | Phase II trial, non-randomized, parallel assignment, open-label | Child, Adult, Older Adult | 48 | June 2014 | Sarcoma Alliance for Research through Collaboration | Completed | No indexed paper found | NCT00304083 |
| Everolimus Plus Bevacizumab | mTOR Inhibitor + VEGF-A inhibitor | Phase II trial, single-arm, open-label | 18 years and older | 25 | December 2017 | Sarcoma Alliance for Research through Collaboration | Completed | With a clinical benefit rate of 12% (3 of 25), there was no objective response in refractory MPNST^38^ | NCT01661283 |
| Ganetespib Plus Sirolimus | Heat shock protein 90 inhibitor + mTOR Inhibitor | Phase I/II trial, single-arm, open-label | 16 years and older | 20 | July 2018 | Sarcoma Alliance for Research through Collaboration | Completed | No objective response^37^ | NCT02008877 |
| **Cutaneous Neurofibromas** | | | | | | |  |  |  |
| Aminolevulinic Acid | Metabolic conversion of ALA to protoporphyrin IX | Phase I trial, single-arm, open-label | 18 years to 90 years | 20 | July 7, 2016 | Medical College of Wisconsin | Completed | TUNEL evaluation showed 42.5 ± 19.9 apoptotic cells per visual field for ALA-treated and 1.1 ± 1.4 for vehicle-treated tumors (p = 0.002)^42^ | NCT01682811 |
| Diclofenac Sodium | Cyclooxygenase inhibitor | Phase II trial, single-arm, open-label | 18 years and older | 7 | June 30, 2017 | Fundação Educacional Serra dos Órgãos | Completed | No significant alterations in terms of presence of tissue necrosis, size, or histopathological features of neurofibromas. No adverse effects were reported^41^ | NCT03090971 |
| Imiquimod 5% | Toll-like receptor agonist | Phase I trial, single-arm, open-label | 18 years and older | 11 | December 2013 | Massachusetts General Hospital | Completed | No posted results on clinicaltrials.gov | NCT00865644 |
| NFX-179 gel | MEK inhibitor | Phase II trial, single-arm, open-label | 18 years and older | 48 | April 14, 2021 | NFlection Therapeutics, Inc. | Completed | Results under review | NCT04435665 |
| NPC-12G Gel 0.2% Sirolimus | mTOR inhibitor | Phase I trial, single-arm, open-label | 18 years to 65 years | 12 | June 17, 2019 | Nobelpharma | Completed | No posted results on clinicaltrials.gov | NCT04461886 |
| Ranibizumab | VEGF-A inhibitor | Phase I trial, single-arm, open-label | 18 years and older | 11 | December 2013 | Massachusetts General Hospital | Completed | No posted results on clinicaltrials.gov | NCT00657202 |
| Talaporfin sodium | Direct cytotoxicity due to reactive oxygen species | Phase I trial, single-arm, open-label | 3 years to 21 years | 7 | July 2012 | Children’s Hospital of Philadelphia | Terminated | No posted results on clinicaltrials.gov | NCT00102115 |
| **Gliomas** | | | | | | | | | |
| Erlotinib Plus Rapamycin (Sirolimus) | EGFR inhibitor + mTOR inhibitor | Phase I trial, single-arm, open-label | up to 21 years | 21 | July 2010 | Roger Packer, Children’s National Research Institute | Completed | No indexed paper found | NCT00901849 |
| Everolimus | mTOR Inhibitor | Phase II trial, single-arm, open-label | 1 year to 21 years | 23 | October 26, 2017 | University of Alabama at Birmingham | Completed | Significant disease stability/shrinkage during treatment^26^ | NCT01158651 |
| Sorafenib | BRAF inhibitor and multiple tyrosine kinase inhibitor | Phase II trial, single-arm, open-label | 2 years and older | 12 | March 2013 | NYU Langone Health | Terminated | Terminated due to toxicity^50^ | NCT01338857 |
| Vinblastine Plus Carboplatin | Microtubule formation inhibitor + Cross-links DNA | Phase I trial, single-arm, open-label | up to 21 years | 26 | March 2012 | Children’s Oncology Group | Completed | No posted results on clinicaltrials.gov | NCT00352495 |
| **Gastrointestinal Stromal Tumor** | | | | | | | | | |
| Selumetinib | MEK inhibitor | Phase II trial, Non-Randomized Parallel Assignment, open-label | 3 years to 99 years | 0 | March 27, 2019 | National Cancer Institute | Withdrawn | Withdrawn (slow accrual) | NCT03109301 |

**Supplementary Table S3. Review of clinical trials in neurofibromatosis type 2-associated tumors and schwannomatosis**

| **Drug** | **Mechanism of action** | **Trial design** | **Age** | **Enrollment** | **Completion or estimated completion date** | **Sponsor** | **Status** | **Results** | **Clinical trial identifier** |
| --- | --- | --- | --- | --- | --- | --- | --- | --- | --- |
| **Vestibular Schwannomas** | | | | | | |  |  |  |
| Aspirin | Cyclooxygenase inhibitor | Phase II prospective, randomized, double-blind, longitudinal study | 12 years and older | 300 | February 2023 | Massachusetts Eye and Ear Infirmary | Recruiting | No posted results on clinicaltrials.gov | NCT03079999 |
| Axitinib | VEGFR inhibitor | Phase II trial, single-arm, open-label | 18 years and older | 13 | February 5, 2019 | NYU Langone Health | Completed | Preliminary results showed that 7 of 12 patients completed 12 cycles with 2 imaging and 3 hearing responses^66^ | NCT02129647 |
| Bevacizumab | VEGF-A inhibitor | Phase II trial, single-arm, open-label | 6 years and older | 22 | February 1, 2020 | University of Alabama at Birmingham | Completed | PR in 32% (7 of 22) of patients. Hearing response in 41% (9 of 22)^12^ | NCT01767792 |
| Bevacizumab | VEGF-A inhibitor | Phase II trial, single-arm, open-label | 12 years and older | 14 | March 2014 | National Cancer Institute | Completed | PR in 43% (6 of 14) of patients. Hearing improvement in 36% (5 of 14)^13^ | NCT01207687 |
| Crizotinib | c-Met and ALK tyrosine kinase inhibitor | Phase II trial, single-arm, open-label | 6 years and older | 19 | December 31, 2025 | University of Alabama at Birmingham | Recruiting | No posted results on clinicaltrials.gov | NCT04283669 |
| Endostatin | VEGF expression inhibitor | Phase II trial, single-arm, open-label | 16 years to 50 years | 20 | April 2016 | Beijing Tiantan Hospital | Completed | No posted results on clinicaltrials.gov | NCT02104323 |
| Everolimus | mTOR inhibitor | Phase II trial, single-arm, open-label | 16 years to 65 years | 4 | February 1, 2023 | Jonsson Comprehensive Cancer Center | Active, not recruiting | No posted results on clinicaltrials.gov | NCT01345136 |
| Everolimus | mTOR inhibitor | Phase II trial, single-arm, open-label | 15 years and older | 10 | January 2017 | Assistance Publique - Hôpitaux de Paris | Completed | No PR in 9 patients. Possible tumor stabilization or growth delay^61^ | NCT01490476 |
| Lapatinib | Tyrosine kinase inhibitor | Phase I trial, single-arm, open-label | 18 years and older | 26 | August 2014 | Sidney Kimmel Comprehensive Cancer Center at Johns Hopkins | Completed | No indexed paper found | NCT00863122 |
| Lapatinib | Tyrosine kinase inhibitor | Phase II trial, single-arm, open-label | 4 years to 80 years | 21 | November 2012 | NYU Langone Health | Completed | PR in 23% (4 of 17) of patients. Hearing improvement in 30% (4 of 13)^62^ | NCT00973739 |
| Nilotinib | Tyrosine kinase inhibitor | Phase II trial, single-arm, open-label | 18 years and older | 2 | October 2013 | University Health Network, Toronto | Terminated | Terminated due to difficulty recruiting | NCT01201538 |
| **Meningiomas** | | | | | | |  |  |  |
| AR-42 (REC-2282) | Histone  deacetylase  inhibitor | Phase II/III  parallel-group, two-staged, randomized | 12 years and older | 89 | July 1, 2027 | Recursion Pharmaceuticals Inc. | Active, not yet recruiting | No posted results on clinicaltrials.gov | NCT05130866 |
| GSK2256098 | FAK Inhibitor | Phase II trial, non-randomized, parallel assignment, four-arm (only one NF2 arm), open-label | 18 years and older | 124 | October 2024 | Alliance for Clinical Trials in Oncology | Recruiting | No posted results on clinicaltrials.gov | NCT02523014 |
| Vistusertib (*AZD2014*) | mTOR inhibitor | Phase II trial, single-arm, open-label | 18 years and older | 18 | October 1, 2020 | Massachusetts General Hospital | Completed | Partial results presented in ASCO meeting: PFS-6 rate that exceeds the RANO target of 35% for recurrent high-grade meningioma^69^ | NCT02831257 |
| **Vestibular Schwannomas and Meningiomas** | | | | | | |  |  |  |
| AR-42 | Histone  deacetylase  inhibitor | Phase I trial, single-arm, open-label | 18 years and older | 5 | October 2023 | Massachusetts Eye and Ear Infirmary | Active, not recruiting | In the postdoc analysis: 6 evaluable patients had 15 tumors (8 VS, and 7 meningiomas). Tumor volume increased in  6, remained stable in 8, and decreased in 1 tumor. There were 10 grade 3 toxicities and 1 grade 4^65^ | NCT02282917 |
| Everolimus (RAD001) | mTOR inhibitor | Phase I trial, single-arm, open-label | 18 years and older | 5 | December 2019 | NYU Langone Health | Completed | No posted results on clinicaltrials.gov | NCT01880749 |
| **NF2-related Tumors** | | | | | | |  |  |  |
| Brigatinib | ALK inhibitor | multi-arm phase II platform-basket screening study | 12 years and older | 80 | December 1, 2030 | Massachusetts General Hospital | Recruiting | No posted results on clinicaltrials.gov | NCT04374305 |
| Everolimus (RAD001) | mTOR inhibitor | Phase II trial, single-arm, open-label | 3 years and older | 10 | December 2013 | NYU Langone Health | Completed | Schwannoma: None of the 9 patients with evaluable disease experienced a clinical or MRI response^60^  Meningioma: 2 of 17 tumors had PD when on treatment versus 8 of 17 off treatment^61^ | NCT01419639 |
| Icotinib | EGFR tyrosine kinase inhibitor | Phase II trial, single-arm, open-label | 16 years to 50 years | 10 | July 2018 | Beijing Tiantan Hospital | Completed | No posted results on clinicaltrials.gov | NCT02934256 |
| PTC299 | VEGF-A inhibitor | Phase II trial, single-arm, open-label | 18 years and older | 11 | March 31, 2012 | Massachusetts General Hospital | Completed | No posted results on clinicaltrials.gov | NCT00911248 |
| Selumetinib | MEK inhibitor | Phase II trial, non-randomized, parallel assignment, open-label | 3 years to 45 years | 34 | May 2024 | Children’s Hospital Medical Center, Cincinnati | Recruiting | No posted results on clinicaltrials.gov | NCT03095248 |
| **Schwannomatosis** | | | | | | |  |  |  |
| Tanezumab | Anti-nerve growth factor (NGF) | Phase II trial, randomized, parallel assignment, open-label | 18 years and older | 46 | June 2024 | Massachusetts General Hospital | Recruiting | No posted results on clinicaltrials.gov | NCT04163419 |
| **Neurofibromatosis type 1, Neurofibromatosis type 2 or Schwannomatosis** | | | | | | |  |  |  |
| Antigen-specific T cells CART/CTL and DCvac | Immunotherapy | Phase I/II trial, randomized, parallel assignment, open-label | 1 year to 80 years | 100 | December 31, 2022 | Shenzhen Geno-Immune Medical Institute | Recruiting | No posted results on clinicaltrials.gov | NCT04085159 |

* Data is not updated on clinicaltrials.gov

** This is the estimated primary completion date. The study completion date was not provided.
